# Ancestral Components of Admixed Genomes in a Mexican Cohort

**DOI:** 10.1371/journal.pgen.1002410

**Published:** 2011-12-15

**Authors:** Nicholas A. Johnson, Marc A. Coram, Mark D. Shriver, Isabelle Romieu, Gregory S. Barsh, Stephanie J. London, Hua Tang

**Affiliations:** 1 Department of Statistics, Stanford University, Stanford, California, United States of America; 2 Department of Health Research and Policy, Stanford University School of Medicine, Stanford, California, United States of America; 3 Department of Anthropology, Pennsylvania State University, University Park, Pennsylvania, United States of America; 4 National Institute of Public Health, Cuernavaca, Mexico; 5 Department of Genetics, Stanford University School of Medicine, Stanford, California, United States of America; 6 HudsonAlpha Institute for Biotechnology, Huntsville, Alabama, United States of America; 7 Epidemiology Branch, National Institute of Environmental Health Sciences, National Institutes of Health, Department of Health and Human Services, Research Triangle Park, North Carolina, United States of America; 8 Laboratory of Respiratory Biology, National Institute of Environmental Health Sciences, National Institutes of Health, Department of Health and Human Services, Research Triangle Park, North Carolina, United States of America; The University of North Carolina at Chapel Hill, United States of America

## Abstract

For most of the world, human genome structure at a population level is shaped by interplay between ancient geographic isolation and more recent demographic shifts, factors that are captured by the concepts of biogeographic ancestry and admixture, respectively. The ancestry of non-admixed individuals can often be traced to a specific population in a precise region, but current approaches for studying admixed individuals generally yield coarse information in which genome ancestry proportions are identified according to continent of origin. Here we introduce a new analytic strategy for this problem that allows fine-grained characterization of admixed individuals with respect to both geographic and genomic coordinates. Ancestry segments from different continents, identified with a probabilistic model, are used to construct and study “virtual genomes” of admixed individuals. We apply this approach to a cohort of 492 parent–offspring trios from Mexico City. The relative contributions from the three continental-level ancestral populations—Africa, Europe, and America—vary substantially between individuals, and the distribution of haplotype block length suggests an admixing time of 10–15 generations. The European and Indigenous American virtual genomes of each Mexican individual can be traced to precise regions within each continent, and they reveal a gradient of Amerindian ancestry between indigenous people of southwestern Mexico and Mayans of the Yucatan Peninsula. This contrasts sharply with the African roots of African Americans, which have been characterized by a uniform mixing of multiple West African populations. We also use the virtual European and Indigenous American genomes to search for the signatures of selection in the ancestral populations, and we identify previously known targets of selection in other populations, as well as new candidate loci. The ability to infer precise ancestral components of admixed genomes will facilitate studies of disease-related phenotypes and will allow new insight into the adaptive and demographic history of indigenous people.

## Author Summary

Admixed individuals, such as African Americans and Latinos, arise from mating between individuals from different continents. Detailed knowledge about the ancestral origin of an admixed population not only provides insight regarding the history of the population itself, but also affords opportunities to study the evolutionary biology of the ancestral populations. Applying novel statistical methods, we analyzed the high-density genotype data of nearly 1,500 Mexican individuals from Mexico City, who are admixed among Indigenous Americans, Europeans, and Africans. The relative contributions from the three continental-level ancestral populations vary substantially between individuals. The European ancestors of these Mexican individuals genetically resemble Southern Europeans, such as the Spaniard and the Portuguese. The Indigenous American ancestry of the Mexicans in our study is largely attributed to the indigenous groups residing in the southwestern region of Mexico, although some individuals have inherited varying degrees of ancestry from the Mayans of the Yucatan Peninsula and other indigenous American populations. A search for signatures of selection, focusing on the parts of the genomes derived from an ancestral population (e.g. Indigenous American), identifies regions in which a genetic variant may have been favored by natural selection in that ancestral population.

## Introduction

During the past decade, data generated by high-throughput genotyping technologies have enabled studies probing into two central questions in human evolutionary biology: the characterization of human population genetic structure, and the search for the molecular signature of natural selection. Insights gleaned from these studies have provided important clues for understanding the phenotypic diversity of our species, and variables representing population structure are routinely incorporated as covariates in genome-wide association studies of complex traits and diseases. At a global level, as well as within a continent or even a sub-continental region, geography has been shown to act as the leading driving force in shaping the pattern of genetic variation that we observe today [1]–[5]. In parallel, analyses based on European, African and East Asian populations have revealed that recent positive selection is a prevalent phenomenon throughout the genome [6]–[8]. Using data from the Human Genome Diversity-CEPH Panel (HGDP), a recent and comprehensive survey suggests that, while adaptation to local environment is a common theme throughout human evolution, the genetic loci involved in adaptation show little overlap among non-contiguous geographic regions [9].

While geography poses a significant reproductive barrier, multiple waves of massive trans-continental migration have occurred during the past centuries, giving rise to admixed populations. The ancestry of non-admixed individuals can often be traced to precise regions based solely on genetic data, but characterizing the sub-continental ancestry origins of an admixed individual has not been demonstrated to date. For example, the two largest minority groups in North America, Latinos and African Americans, both arose as a result of mating among populations that had been in historical reproductive isolation. The “Hispanic” or “Latino” populations include the ethnically diverse groups of Latin America; although significant genetic contributions can be traced to Indigenous American, European and West African populations, it has been challenging to determine whether one's Indigenous American ancestors originate from North, Central, or South America. Solving this problem has implications for both a deeper understanding of human evolution and for human disease, since genetic diversity between Latino populations is characterized both by variation in continent-level ancestry – e.g. Mexicans on average have lower African ancestry than Puerto Ricans – and by the population structure among the ancestral Indigenous American populations [4], [10].

The assessment of the precise ancestral origin and the quantification of genetic structure within an ancestry component are limited, in part, by analytic challenges. Principal Component Analysis (PCA) is a classic technique for multivariate data analysis, which aims to project high-dimensional data to a much lower dimension while capturing the greatest level of variation [11]. This approach has gained popularity in genetic analyses due to both computational efficiency and interpretability: when the underlying population structure is driven mainly by reproductive isolation and subsequent genetic differentiation, the principal components (PCs) mirror the geographic origins of individuals [3]. By itself, however, PCA is not well suited for studying admixed populations: while leading PCs usually represent the relative contributions of continentally-divided ancestral populations, subsequent PCs may be simultaneously influenced by structures within one or more of the ancestral populations, and are consequently difficult to interpret.

We tackled this problem by employing an analytic strategy that works backwards according to the temporal nature of demographic events that underlie human admixture: genomes are first separated into the major and most recent components that reflect inter-continental migration, then each of those components is further investigated separately. As described below, we apply a probabilistic method for inferring locus-specific ancestry along the chromosome, followed by a variant of PCA to further investigate each of the ancestry-specific genomic components, which we term “virtual genomes”. This hierarchical strategy yields a fine-scale view of genetic structure in admixed populations, and provides insight into the population history of nonextant ancestral populations. As an example, we study a cohort of 492 parent-offspring trios recruited from Mexico City. Our results confirm the a priori expectation that the most significant European contributors to the Mexican gene pool are populations from the Iberian Peninsula, but reveal that the Indigenous American component of the Mexican genomes is more complex.

Studying the genetic structure of admixed genomes also offers the unique opportunity to probe the adaptive landscape of the ancestral populations. This is particularly powerful for studying the Indigenous American populations, for which limited genotype data is available. As proof of principle, we report a novel application of the extended haplotype homozygosity test for recent positive selection to the European and Indigenous American “virtual genomes” evident in the Mexican cohort, and identify numerous loci as potential targets of positive selection.

## Results

### Overview

**Figure 1:**
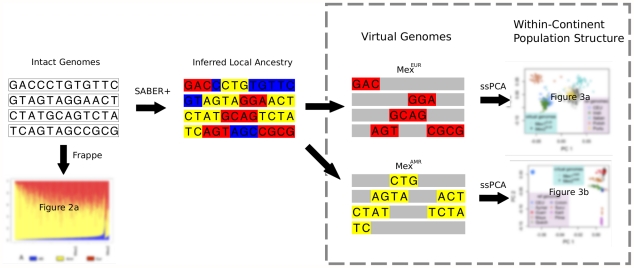
Schematic describing the analytic framework for characterizing continental-level and within-continent populations structure in an admixed population. For virtual genomes, haplotypes from all but one ancestral population are “masked” as missing data.

Our analytic strategy for studying population structure in admixed populations is shown in Figure 1; details of the approach are described in what follows, and in the Materials and Methods section. This approach first applies a model-based clustering method, *frappe*, to the intact genotype matrix, identifying components that correspond to variation in continental-level admixture proportions, and estimating the relative proportion of those components for each individual. Locus-specific continental ancestry along a genome is then inferred using SABER+, an extension of a Markov-Hidden Markov Model method [12] that partitions each genome into ancestral haplotype segments or “virtual genomes”. Finally, within-continent population structure is determined by applying PCA to the virtual genomes, treating the rest of the genome as missing. To account for the large amount of the missing data resulted from the continent-specific genomes, we implement a variation of the subspace PCA (ssPCA) algorithm [13].

Most of the results described here are from a panel of 492 Mexican parent-offspring trios recruited from Mexico City (MEX1) as part of a previous genome-wide association study using genotype data from the Illumina 550K platform [14]. For comparison, we also examined data from 23 HapMap Phase3 Mexican trios recruited from Los Angeles, California (MEX2; http://hapmap.org). Reference populations for inferring continental-level ancestry were taken from HapMap (CEU, YRI), and additional sources as described below and in Table S1.

### Continental-level ancestry: Global and local estimates

**Figure 2:**
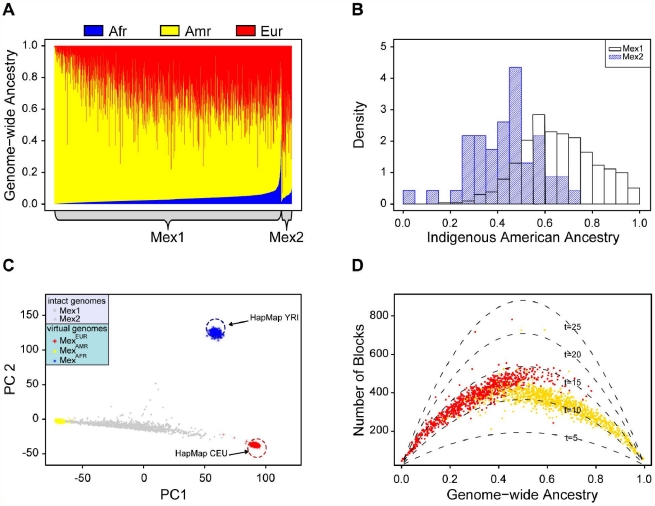
Continental-level ancestry proportions and admixing time. (A) Individual ancestry proportions (red = European; yellow = Indigenous American, blue = African). (B) Histogram comparing the Indigenous American ancestry in the Mexico City cohort (Mex1) and Los Angeles cohort (Mex2). (C) Principal component analysis of the Mexican individuals from Mexico City and Los Angeles, CA. Red and blue circles indicate the location of the HapMap CEU and YRI individuals, respectively. Gray points represent admixed individuals; virtual genomes, Mex^AMR^ (yellow), Mex^EUR^ (blue) and Mex^EUR^ (red) are projected to the PC plot. (D) Admixing times estimated by the number of ancestry blocks. The number of European (vs non-European) ancestry blocks is plotted against European ancestry (red); analogously, the numbers of Indigenous American (vs non-Indigenous American) blocks are plotted against the Indigenous American ancestry (yellow). Each curve represents the expected number of such blocks for a specific admixing time (in generations).

Among the 984 parents of the Mexico City trios (MEX1), we used *frappe* to estimate median ancestry proportions of 65% Indigenous American, 31% European, and 3% African; the corresponding statistics in the 46 HapMap Mexican individuals from Los Angeles (MEX2) are 45%, 49%, and 5%, respectively (Figure 2A). The distribution of Indigenous American ancestry in the Mexico City population is shifted upward compared to the Los Angeles population (Figure 2B), which may reflect differences in the extent of European admixture. African ancestry is low in both cohorts, although the distribution is skewed to the right, reaching over 40% for some individuals.

We next used SABER+ to estimate recombination breakpoints between ancestral chromosomes and thus locus-specific ancestral origin— Indigenous American, European, or African—in individuals from the MEX1 and MEX2 cohorts. For the work described here, the primary goal of SABER+ is to partition the Mexican genomes into haplotype segments according to continental ancestry that can be used for subsequent analysis. However, the output of SABER+ can also be used as an independent means of assessing global ancestry, simply by averaging locus-specific ancestries across all markers, and yields estimates that are highly correlated (r>0.99) with *frappe* (Figure S1).

To facilitate the analyses of sub-continental genetic structure, we constructed *virtual genomes* by retaining haplotype segments from a single continental-ancestral population, while masking (i.e. setting to missing) segments from all other ancestral populations; for example, MEX1^AMR^ and MEX1^EUR^ denote the sets of Indigenous American and European haplotype segments from the Mexico City individuals, respectively.

In a principal component analysis of this data that includes the YRI and CEU HapMap populations, the Indigenous American, European, and African virtual genomes mark vertices of a triangle (Figure 2C) in which the intact genomes of the MEX1 and MEX2 individuals are distributed broadly along an Indigenous American – European axis represented by PC1. The exact position of the intact MEX1 and MEX2 genomes depends on admixture proportions; individuals with the greatest level of African ancestry, which corresponds to PC2, mostly lie at intermediate positions along the Indigenous American -European axis. Importantly, the MEX^EUR^ and MEX^AFR^ virtual genomes (red and blue points, respectively) form discrete clusters whose locations coincide with those of the HapMap CEU and YRI, respectively, and, while there is no reference population in this analysis for Indigenous American, the MEX^AMR^ virtual genomes also form a discrete cluster at a vertex of the triangle. These observations suggest that the ability of SABER+ to assign local ancestry to a specific continental origin is highly accurate, which is essential for subsequent analyses.

### History of admixture

The distribution of the length of ancestry blocks is shaped by population history since admixture. When two individuals from different parental populations mate, the first generation offspring inherits exactly one chromosome from each parental population. In subsequent generations, recombination events in an admixed individual generate mosaic chromosomes of smaller ancestry segments. Intuitively, more recent admixing gives rise to longer ancestry blocks than older admixture. Furthermore, conditioning on the time since admixing within an individual's pedigree, block length distribution also depends on the individual level ancestry proportions: e.g., an individual with 90% European ancestry tends to have long European ancestral blocks because recombination events in the person's genealogy are likely to have joined two European haplotypes, and therefore fewer ancestry changes are expected.

A likelihood-based model has been proposed that can estimate several aspects of admixture history [15]. However, the admixing rates in Mexicans from the European, Indigenous American and African ancestral populations are likely dependent and difficult to model with this likelihood-based method; therefore, we attempted to estimate admixing time using a different approach. We first computed the theoretical number of ancestry blocks for individuals according to their ancestral proportions, and carried out that computation assuming a series of different admixing times (5–25 generations, dotted lines in Figure 2D). The parabolic shape of these curves conforms to the intuitive idea outlined above that the number of block peaks at an intermediate ancestry proportion.

We then superimposed the observed number of ancestry blocks in each MEX1 individual onto the theoretical curves; these results suggest an admixing time of 10–15 generations ago (Figure 2D). The admixing time of the European component appears slightly longer than that for the Indigenous American component (15 generations vs. 12); one potential explanation is that some mixing occurred between the European and the African ancestral individuals prior to admixing with the Indigenous American populations.

### Regional ancestry of virtual genomes

With the MEX^EUR^ uncoupled from the MEX^AMR^ genomes, we investigated structure within each of these virtual genomes separately. (We did not investigate the MEX^AFR^ virtual genomes due to their small sample size). Because there is a large amount of missing data, e.g. the virtual genome of one individual may cover very different loci from the virtual genome of other individuals, we used the ssPCA approach as described in Materials and Methods. To help evaluate the robustness of our approach, we carried out simulation experiments, in which the effects of random error in the inference of continental locus-specific ancestry were measured with regard to their impact on accuracy of within-continent substructure estimates. Results summarized in Materials and Methods and Figure S2 indicate that European substructure can be well separated in the presence of up to 5% error, i.e. Indigenous American alleles mistakenly included in the European virtual genomes, which is well above the level of uncertainty (<2%) associated with the SABER+ approach.

**Figure 3:**
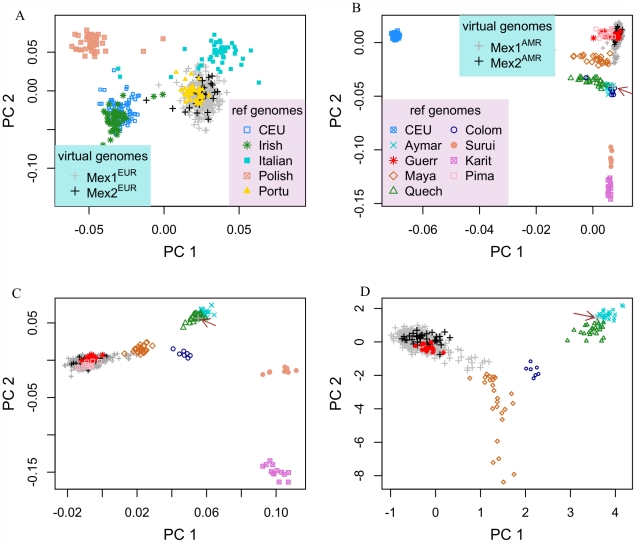
Population structure within the European and Indigenous American components of Mexican genomes. (A) Principal component analysis of the European chromosomal segments traces the ancestry origin closest to Portuguese. (B) Principal component analysis of Indigenous American segments, HapMap CEU and various Indigenous American populations. (C) Same as (B), with CEU individuals removed. (D) Same as (B), with CEU, Surui, Karitiana and Pima removed. The arrow highlights a Mexican individual whose ancestry is traced to the South American group of Aymara.

In addition to the HapMap CEU, who are mostly of Northern European ancestry, we used individuals recruited from Dublin, (Ireland), Warsaw (Poland), Rome (Italy) and Porto (Portugal) to provide references for different areas within Europe. The first two PCs provide good separation of these reference populations, and correspond roughly to North-South and West-East gradients (Figure 3A). Both the MEX1^EUR^ and MEX2^EUR^ virtual genomes are most closely related to intact genomes from Porto, which we interpret as a surrogate for populations from the Iberian Peninsula, [3], consistent with the historical record that the first European migrants to Mexico were Spaniards.

For analysis of the MEX^AMR^ virtual genomes, we introduced 129 individuals representing 8 different Indigenous American populations as reference genomes (Table S1) [16]. Initially, we also included the HapMap CEU based on previous results in which some Indigenous American individuals from the Human Genome Diversity Panel (HGDP) were observed to have non-negligible levels of European ancestry [2]. Indeed, the first two PCs for this analysis occur along European-Indigenous American and within-America axes (Figure 3B), and reveal varying levels of European ancestry in the Mayan, Quechua and Colombian populations. In this analysis and subsequent ones carried out in which certain reference populations were removed (CEU removed from Figure 3C; CEU, Surui, Karitiana and Pima removed from Figure 3D), the MEX^AMR^ virtual genomes are most closely related to intact genomes of individuals from southwestern Mexican state of Guerrero (Guerr), which includes Nahua, Mixtec and Tlapanec indigenous groups. Although the Guerrero individuals and the Pima individuals cluster together in Figure 3C, they are separable on PC 3 (Figure S3), along which the Guerrero, but not Pima individuals, cluster with MEX^AMR^. The Indigenous American virtual genomes of Mexicans from Mexico City (MEX1^AMR^) are similar to those from Los Angeles (MEX2^AMR^); further, we observe a gradient with varying contribution from Mayans, with some Mexicans deriving their Indigenous American ancestry predominantly from Mayans. One individual from Mexico City has an Indigenous American virtual genome that is localized with the Quechua (arrow, Figure 3C) and therefore is likely to have a source of Indigenous American ancestry that is distinct from that of the other Mexicans.

### Investigating natural selection in ancestors of admixed genomes

The ability to accurately construct ancestral virtual genomes from admixed genomes provides a number of opportunities in the areas of human evolution and genetic anthropology. As an example of how such data can be used more generally, we examined the Mexican ancestral components for regions of extended haplotype homozygosity, which mark loci that have undergone recent positive selection. We used the integrated haplotype score (iHS) statistic [8], with a modified normalization procedure so as to fit a standard normal distribution.

**Figure 4:**
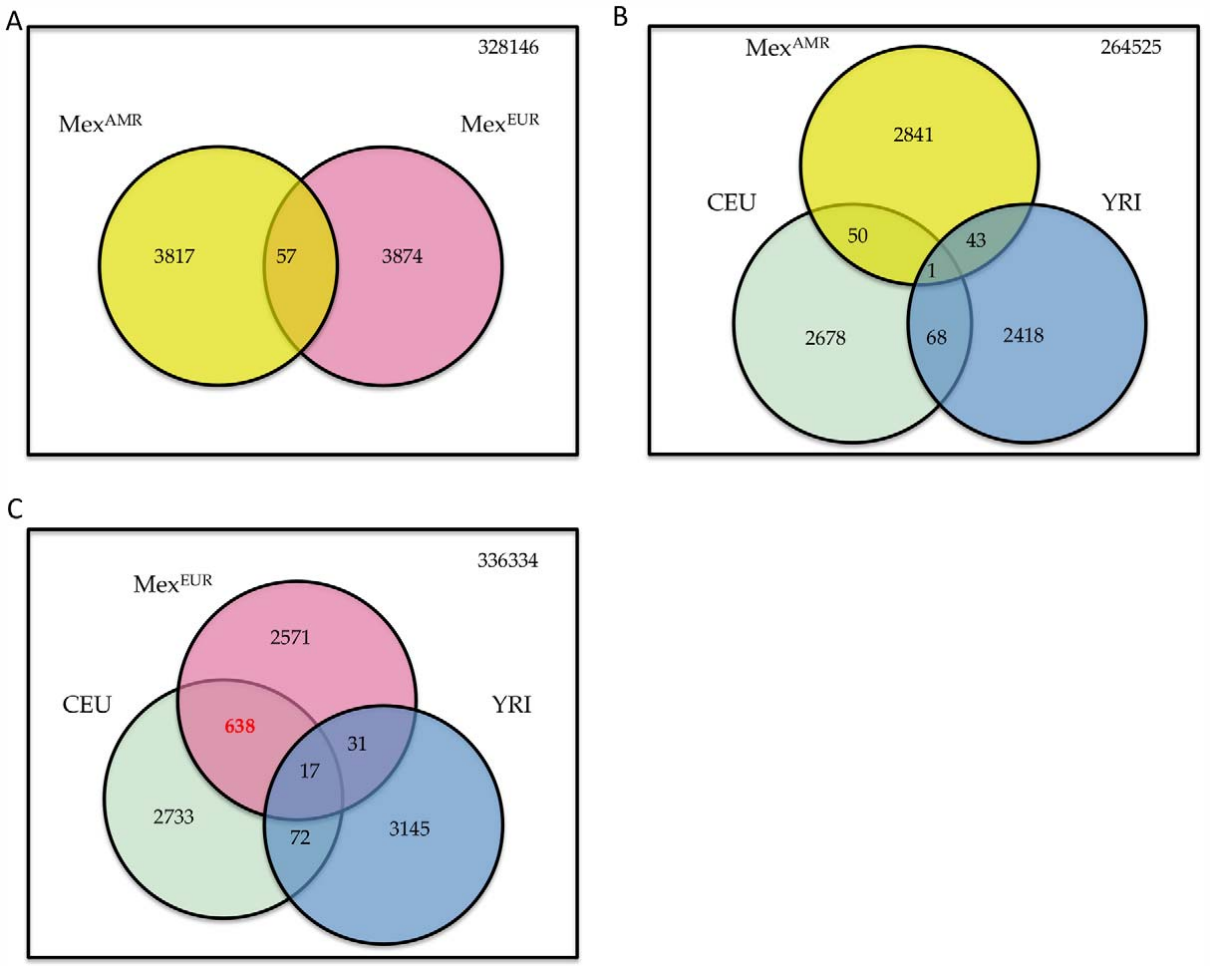
Signatures of recent positive selection. (A) Overlap of top 1% SNPs (with |iHS|>2.5) in Mex^EUR^ and Mex^AMR^. (B) Overlap of SNPs with |iHS|>2.5 in Mex^AMR^, HapMap CEU and YRI [8]. (C) Overlap of SNPs with |iHS|>2.5 in Mex^EUR^, HapMap CEU and YRI. Numbers in red denotes significant enrichment in overlap.

For the virtual genome SNPs that show the strongest evidence of positive selection, the degree of overlap between the Europeans and Indigenous Americans is similar to that expected by chance (Figure 4A). Specifically, we considered SNPs with |iHS|>2.5, which represent approximately the top 1% scores in either components; 3874 and 3931 SNPs meet this criterion in MEX^AMR^ and MEX^EUR^, respectively, with 57 SNPs overlap between the two sets (expected overlap = 40, p = 0.094). Similarly, we found little overlap between the iHS scores in MEX^AMR^ and those computed based on the HapMap populations [8] (Figure 4B). In contrast, the correlation is much higher between the iHS in MEX^EUR^ and those from HapMap CEU (r = 0.79), which reflects shared population and adaptive histories of Southern Europeans (the MEX^EUR^) and the CEU (mostly from Northern and Central Europe). Specifically, of 3257 and 3460 SNPs with |iHS|>2.5 in MEX^EUR^ and CEU, respectively, 655 are overlapping (expected overlap = 32, p<2.2^−16^) (Figure 4C). These findings are consistent with previous observations that intact genomes from the HGDP collection exhibit histories of positive selection that differ according to continent [9].

We also asked what genes might underlie the strongest signatures of positive selection. Towards this end, we grouped SNPs into 50kb windows, selected regions with at least 20 SNPs and at least 10% of SNPs with |iHS|>2.5, and ranked windows by the maximum |iHS| score. The top 10 regions within the MEX^EUR^ and MEX^AMR^ components are shown in Table 1 and Table 2. The only genomic location that features in both lists is the HLA region on chr 6p, a region known to have experienced strong selection [17]; however, the precise variants that show high iHS scores differ between the Europeans and Indigenous Americans. Outside the HLA region, the most prominent signal in the European component coincides with APBA2 on chr 15q, which is in close proximity to a known pigmentation gene, OCA2. In the Indigenous Americans component, the strongest signal occurs in chr 6p12.3-2; this region harbors numerous genes, including IL17A which is associated with chronic inflammatory diseases such as rheumatoid arthritis, and PKHD1 which is associated with polycystic kidney disease [18], [19].

**Table 1:** Regions showing strongest evidence of recent positive selection in the European component.

Cytological Position	Genes (Number)	Size(kb)	Number of SNPs with |iHS|>2.5	Max |iHS|
6p21.33-32	TRIM31, HLA (88)	2000	113/581	5.71
15q11-q12	APBA2 (1)	194	30/37	4.41
17q24	RGS9 (1)	268	33/54	4.36
18p11.23	PTPRM (1)	192	24/34	4.31
15q26.1	none	154	20/50	4.02
5q22.1	TMEM232, SLC25A46 (2)	309	19/26	4.00
9p22.3	SMARCA2 (1)	87	29/47	3.95
3q22.1	COL6A5 (1)	164	17/36	3.89
9q33.2	DENND1A (1)	480	34/61	3.86
3p14.1	none	82	13/23	3.84
22q12.2	SFI1, PISD (3)	267	17/31	3.83

Gene (Number) displays the number of genes in the region; in the case a region encompasses multiple genes, we arbitrarily choose two. Size denotes the length of the region under selection; consecutive regions are merged. Number of SNPs with |iHS|>2.5 provides the number of SNPs with |iHS|>2.5 / total number of SNPs. Max |iHS| is the maximum |iHS| score achieved in a region.

**Table 2:** Regions showing strongest evidence of recent positive selection in the Indigenous American component.

Cytological Position	Genes (Number)	Size(kb)	Number of SNPs with |iHS|>2.5	Max |iHS|
6p21.33	TRIM40, TRIM31 (4)	75	4/22	4.33
6p12.3-2	IL17, PKHD1 (18)	3083	181/333	4.26
19q13.11	SLC7A9, GPATC1 (6)	421	37/59	4.23
12p12.1-23	SSPN, ITPR2 (2)	547	70/123	4.15
1q24.1	MAEL, GPA33 (3)	164	24/45	4.14
10p12.32	PLXDC2 (1)	123	11/40	3.94
14q12	STXBP6 (1)	146	13/30	3.92
16q23.2	none	197	17/48	3.72
5q33.1	SLC36A3, GM2A(3)	133	14/29	3.54
2q24.2	LY75, PLA2R1 (2)	244	18/53	3.53

Gene (Number) displays the number of genes in the region; in the case a region encompasses multiple genes, we arbitrarily choose two. Size denotes the length of the region under selection; consecutive regions are merged. Number of SNPs with |iHS|>2.5 provides the number of SNPs with |iHS|>2.5 / total number of SNPs. Max |iHS| is the maximum |iHS| score achieved in a region.

## Discussion

Previous approaches to analyzing the ancestry in admixed individuals have largely focused on estimating continental-level admixture proportions. Within continental ancestry analyses have been performed at a population level but not at an individual level. Our approach is distinct in several ways from a recent study, which reports the affinity, at a population level, of admixed populations to various ancestral groups [20]. This latter approach requires a pre-defined notion of subpopulation, such as Mexicans versus Puerto Ricans, and produces a population level summary of genetic relationship. In contrast, our approach does not rely on such pre-defined ethnic groups, and thus has the ability to identify previously unrecognized substructure at an individual level, such as the detection of one individual with South American ancestry. In what follows, we first discuss aspects of the approach that may be generally relevant, and then provide some insights in the evolutionary history of the Mexican population. In the context of genome-wide association studies of complex traits and diseases, variables representing both continental-level and within-continent population structure need to be adjusted to provide a more accurate correction for population stratification [21], [22].

Two methodological innovations contributed to the hierarchical depiction of individual ancestry origin: an improved algorithm for locus-specific ancestry inference, which accommodates multiple ancestral populations, and a subspace PCA algorithm that permits varying degrees of missing data. SABER+ uses a graphical model to account for haplotype structure within an ancestral population, and is more accurate for analyzing high-density genotype data. The accuracy of the locus-specific ancestry is supported by two observations. First, in the continental-level PCA analysis (Figure 1A), all “virtual genomes” that are attributed to a single ancestral population, cluster tightly with the reference individuals. Had there been substantial error in the local ancestry inference, some of these genomes would appear admixed and lie in between the vertices. Second, we included the HapMap CEU individuals in the analysis of the Indigenous American components of the genomes because some of the Mayan individuals have been shown to have European admixture [2]. Indeed, although Figure 3B clearly reveals European admixture in some Mayan and Quechua individuals, little European admixture is detected in the putative Indigenous American genomes of the Mexicans, Mex^AMR^. We note that, although many methods for estimating local ancestry, including the method used in this study, are applicable to unphased data, the parent-offspring trio structure of the Mexican data (both the Mexico City cohort and the HapMap Mexican sample) allows accurate haplotype phasing for each individual, which likely improves the accuracy of inference of local ancestry along each haplotype. As a result, both phasing and ancestry inference are likely more accurate than those estimated based on unphased genotype data.

Typically, application of PCA for genetic structure analyses makes use of the program Eigenstrat, which is based on the eigen-decomposition of the covariance matrix, 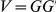, where *G′* is the transpose of the centered and scaled genotype matrix, *G* [21]. In computing this covariance matrix, missing genotypes are set to the column means; thus *V_ij_* is computed based on genotypes that are non-missing in both individuals *i* and *j*. While this approach is adequate for analyses based on high-density genotypes with very low levels of missing genotypes, it is not appropriate for analyzing the virtual genomes, which feature large and varying proportions of missing data. Consider two individuals each with 30% ancestry from the population of interest (e.g. Europe): within each individual, 9% of the genome is expected to be homozygous in European ancestry, and therefore <1% of the markers are expected to be non-missing in both genomes after excluding non-European genotypes. This leads to two problems: first, it has reduced power for detecting population substructure because it uses only a small fraction of informative genotypes for the continent of interest; more importantly, the sampling variability of the covariance estimates depends heavily on the proportion of missing genotypes, biasing the PCs such that individuals with high missing rate become outliers along each PC. The ssPCA we implement does not require the computation of the covariance matrix, and uses all informative markers in each genome; hence it is less sensitive to the missing data. Since the algorithm can compute the first *k* PCs without computing all PCs, it also has computational advantages over the current covariance-based implementation, especially when the number of individuals is large.

### Pattern of ancestry origin

We find extensive variation with respect to continental-level ancestry proportions, both between geographic regions – shown by the much higher Indigenous American ancestry in the Mexico City cohort compared to the HapMap Mexican Americans from Los Angeles – and between individuals within each cohort. This study benefits from the ability to divide the genome of a single Mexican individual into its constituent ancestral components. The ability to trace chromosomal segments to their respective ancestral populations allows us to scrutinize the ancestry origin of each individual within a continent. Within the European component of the Mexican genomes (Mex^EUR^), nearly all individuals, both from Mexico City and from Los Angeles, trace their European ancestries to a Southern European population, as represented in our study by the Portuguese. Within the Indigenous American component of the genomes (Mex^AMR^), a majority of individuals trace their ancestries to groups from the southwest coastal regions of Mexico, consistent with a previous study, which found Zapotec individuals from the State of Oaxaca to best approximate the Indigenous American ancestral population for Mestizos [23]. Importantly, we find evidence of varying levels of Mayan admixture, as well as one individual with Indigenous American ancestry from Bolivia/Peru. Of note, individuals with high levels of Mayan or South American ancestries do not stand out in the continental-level PCA, as their continental-level ancestry proportions are comparable to the rest of the Mexicans.

The finding that most Mexican individuals trace their European and Indigenous American ancestry to well-defined geographic regions contrasts sharply the lack of structure in the African ancestry in the African Americans: not only did we trace each African American individual to multiple West/Central West African groups, but the relative proportions are nearly constant across all individuals [24]. This difference can be reconciled by the distinct migratory histories: the African ancestry in African-American populations is largely derived from the trans-Atlantic slave trade, which forcibly departed African individuals from various geographic regions of Western Africa, ranging from Senegal to Nigeria to Angola [25]. In contrast, no evidence suggests massive relocation of the Indigenous Americans during the colonization in North America, and hence reproductive isolation likely has been maintained between geographically separated Indigenous American populations. One limitation of the current study is the incomplete sampling of the Indigenous American populations in our reference panel, which represents two distinct regions in Mexico: the Southwest coastal State of Guerrero and the Yucatan Peninsula. Thus, while most Mexicans trace their Indigenous American ancestries to the indigenous groups from the State of Guerrero (Guerr), it is possible that the true ancestors of the extant Mexicans are an un-sampled group that is genetically similar to Guerr. With the coming of whole genome sequencing data, it is possible that indigenous populations from neighboring states can be distinguished, and thus it may even be possible to detect admixture from closely related Indigenous American groups.

### Selection

The EHH analyses of the Southern European and the Indigenous American components of the Mexican genomes separately revealed numerous intriguing putative targets of recent positive selection. We note that many other approaches have been developed to detect specific types of selective events, and are equally applicable [26], [27]. We chose to use the iHS test because it has been applied to both the HapMap dataset and the HGDP dataset, thus facilitating comparison. The goal of this paper is not to conduct a comprehensive survey of the selective landscape in the ancestral populations of the present day Mexicans, but rather to illustrate the potential benefits of such endeavors. Given the difficulties in recruiting large samples of non-admixed indigenous individuals from each well-defined Indigenous American group, we argue that admixed populations will provide valuable insight in future endeavors in understanding the evolutionary histories of the Indigenous American populations, some of which may have been extinct. For example, individuals with full Taíno ancestry are rare, but approximately 15% of the contemporary gene pool of Puerto Ricans may have been derived from Taínos. Hence, admixed Puerto Rican genomes can be used to learn about those of the ancestral Taínos [28]. We note that this approach of assembling an ancestral population from a mixed population has also provided important insights in the Aboriginal Australian population in a recent study [29].

Distinguishing between selective events that occurred within the ancestral populations and those that occurred post-admixing requires careful consideration of the tests and associated assumptions. In the current setting, we reasoned that, since a novel adaptive allele is unlikely to be swept to a substantial frequency within a period of less than 500 years (since the arrival of the Europeans in Mexico), and since the EHH method does not have appreciable power to detect low frequency adaptive alleles [9], most of the signals detected by the EHH had occurred prior to admixing, and hence represent selection within the ancestral populations. On the other hand, the preservation of a long haplotype excludes the possibility of very ancient selective events; this belief is also supported by the observation that there is little overlap between the signatures detected in the Southern European and the Indigenous American components. In previous studies of Puerto Ricans and African Americans, numerous genomic locations were found where locus-specific ancestry deviate from the genome-wide average, and could represent targets of selection in the admixed populations [30], [31]. In the current analyses, the only locus showing deviation from the genome-wide average is the HLA region on chr 6, again supporting a population-specific pattern of selection. Therefore, the adaptive history of the Indigenous American groups may vary considerably, and should be studied separately and not as a whole group. Such analyses can be achieved, for example, by examining the Indigenous American components in Mexicans versus that of Puerto Ricans.

Our results have important implications for the design of genome-wide association studies based on admixed populations. Epidemiologic studies have found varying prevalence of conditions such as asthma, diabetes and alcohol-related problems across Hispanic national groups [28], [32], [33]. Distinct population and adaptive history among Hispanics ethnic groups can give rise to heterogeneity in complex traits. Therefore, the importance of accounting for intra-continental genetic structure in disease mapping studies, in addition to adjusting inter-continental admixture proportions, needs to be carefully evaluated.

## Materials and Methods

### Populations

The Mexican individuals analyzed in this project come from two sources: a panel of 492 Mexican parent-offspring trios recruited from Mexico City as part of a previous genome-wide association study (MEX1) [14], and 23 HapMap Phase3 Mexican trios recruited from Los Angeles, California (MEX2; http://hapmap.org). For estimating locus-specific ancestry, we used the HapMap CEU (N = 88) and YRI (N = 100) individuals for the ancestral populations. To analyze the European component of the admixed genome, we augmented the Mexican datasets with individuals recruited from Dublin, Ireland (N = 43), Rome, Italy (45), Warsaw, Poland (N = 45) and Porto, Portugal (N = 43). For the Indigenous American component analyses, we combined the data generated in two previous studies [2], [16]. Four Mayan individuals with substantial European admixture are removed. The combined set used for the subsequent analyses includes 14 individuals from Guerrero, Mexico (two Nahua, seven Mixtec and five Tlapanec), 24 Mayan individuals from the Yucatan Peninsula, 24 Quechua collected in Cerro de Pasco, Peru, 25 individuals of largely Aymara ancestry collected in La Paz, Bolivia, 13 Karitiana and eight Surui from Brazil, seven Colombians, and 14 Pima. Because the sample sizes for Nahua, Mixtec and Tlapnec are small, and all individuals were recruited from the same state, we considered these individuals as one group. Table S1 summarizes the individuals used for each analysis.

### Genotyping QC and haplotype construction

Genotyping and quality control procedures have been described in the primary publications for each dataset, except for the dataset of 176 European individuals. Briefly, MEX1 and the HGDP individuals were genotyped on Illumina 550K and on 650K Beadchip, respectively. The Indigenous American individuals from Bigham et al. (2009) were genotyped on Affymetrix 1M SNP arrays [16]. The set of 176 European individuals were genotyped using Illumina HumanHap300 arrays; this dataset originally included 180 individuals; four individuals were found with non-negligible non-European ancestry and were excluded. SNPs with a call rate of less than 95% were excluded. The number of individuals and markers used for each analysis is summarized in Table S1. We used BEAGLE to construct haplotypes for Mexican trios [34]. As children provide no additional information regarding population structure or adaptation, they are not used in subsequent analyses.

### Genome-wide and locus-specific ancestry inference

Continental-level admixture proportions were estimated two ways: (1) a model-based clustering algorithm implemented in *frappe* [35], and (2) average locus-specific ancestries across all markers. Locus-specific ancestry was estimated with SABER+, an extension of a previously described approach, SABER, that uses a Markov-Hidden Markov Model [12]. SABER+ differs from SABER in implementation of a new algorithm, an Autoregressive Hidden Markov Model (ARHMM), in which haplotype structure within the ancestral populations is adaptively constructed using a binary decision tree based on as many as 15 markers, and which therefore does not require a priori knowledge of genome-wide ancestry proportions (Johnson et al., in preparation). In simulation studies, the ARHMM achieves accuracy comparable to HapMix [36] but is more flexible in modeling the three-way admixture in the Mexican population and does not require information about the recombination rate.

HapMap CEU and YRI individuals were used as the reference ancestral populations. Based on *frappe* and supported by PCA, 50 individuals in MEX1 set have more than 95% Indigenous American ancestry. These individuals were initially used to approximate the Indigenous American ancestors in the locus-specific ancestry analyses; an iterative procedure is used to identify and correct for the non-Indigenous American segments in these individuals. Accuracy of the locus-specific ancestry is verified by performing a PC analysis, treating each individual as three non-admixed genomes, Mex^EUR^, Mex^AMR^, and Mex^AFR^ (see section “subspace PCA” below).

### Subspace PCA (ssPCA)

We implemented this algorithm to accommodate the large amount of missing genotype data in partially masked virtual genomes, and used it to derive all the PCA results reported here. The statistical theory of the algorithm in a general data mining context can be found in [13]; however, various modifications are required for the current setting, as described below. Let ***G^h^*** (h = 1,2) be two *N*×*M* matrices, in which 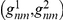 denote the unordered pair of alleles at SNP *m* (*m* = *1*,…,*M*) in individual *n* (*n* = *1*,…,*N*); the columns of ***G^h^*** are standardized to have mean 0 and variance 1. To compute the subspace spanned by the first *k* principal components (PC), we begin by finding a matrix decomposition, 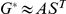, which minimizes the reconstruction error, *R*, defined as:
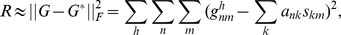
subject to the constraints that the column vectors of **A** are of unit norm and mutually orthogonal and the row vectors of **S** are also mutually orthogonal. Here **A** is a *N*×*d* matrix, ***S*** is a *M*×*d* matrix, and *d<N≤M* represents the desired number of leading PC's. The algorithm we use is a generalized instance of the coordinate descent approach [37], which iteratively optimizes matrix ***A*** for fixed ***S*** and then optimizes ***S*** fixing ***A*** according to the rules:
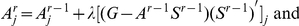


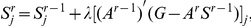
where *λ* is a learning rate, the superscripts, *r*, indicate iteration, and the subscripts, *j*, denote the *j*-th column of a matrix. It can be shown that the columns of ***A*** and ***S*** span the subspace of the first *d* PCs, and that the leading PCs can be computed by orthogonalizing the columns of ***A*** and ***S*** [13]. To evaluate the accuracy of our modified ssPCA approach, we applied it in parallel with Eigenstrat [21] to the intact Mexican genomes, and found the leading PCs produced by the two algorithms were virtually identical, up to a permutation of signs.

### Simulation study with known substructure

We carried out two simulation experiments to evaluate the impact of statistical uncertainties associated with estimating locus-specific ancestry, and to investigate the performance of the ssPCA approach.

In the first set of simulations, we created 10 datasets in which 400 admixed genomes were modeled to mimic a Latino population: each individual draws chromosomal segments from European and Indigenous American ancestry, and the proportion of Indigenous American ancestry in each individual matches what we observed in MEX1. For 200 individuals, European-derived segments were sampled from the HapMap CEU haplotypes, representing Northern and Western European ancestry, while for the remaining 200 individuals, the European-derived segments were sampled from Mex^EUR^ inferred from the actual Mexican genotype data, representing Southern European ancestry. The chromosomal segments from CEU and Mex^EUR^ in the admixed individuals were treated as the true European virtual genomes.

To evaluate the potential impact of statistical uncertainty, we introduced random errors in which the true identities of European vs. Indigenous American segments were switched with probability *ε*. The top PC for each set of simulated virtual genomes (at each of 8 error rates, *ε* = 0.01–0.20) was computed with ssPCA. We evaluate the effect of these errors by calculating a confusion fraction, *ξ*, that quantifies the accuracy with which the estimated first PC separates individuals with Northern vs. Southern European ancestry, and is defined as the proportion of individuals that lie on the “wrong” side of a threshold that best separates the two groups. Thus, *ξ* can range from 0 (perfect separation) to nearly 50% (complete confusion as would be observed for genetically homogenous groups). Finally, we analyze each of the 10 datasets using SABER+, exactly as was done for real data: apply ssPCA to estimate substructure, and calculate a confusion fraction. The results for this simulation experiment are depicted in Figure S2, and show that the confusion fraction increases substantially, from a mean of 2.14% to 17.5%, at error rates between 0.03 and 0.05. Using SABER+ on these same 10 datasets yields a mean confusion fraction of 1.58%, which corresponds to an error rate <0.02 (indicated by the arrow in Figure S2).

In a second set of simulations to investigate the ability of the ssPCA approach to deal with missing data, we created five datasets in which the proportion of genome-wide European ancestry in each of 400 admixed genomes was fixed at either 50% or 30%, respectively. Applying SABER+ and ssPCA to these datasets yields mean confusion fractions of 0 and 0.7%, respectively, indicating that our approach performs well for situations such as the one described here, where mean genome-wide continental ancestry proportions are above 30% for both the European and the Indigenous American components.

### Estimating time since admixing

We used the number of ancestry blocks in an individual as summary statistics. Tracing through a pedigree of *T* generations, the expected number of recombination events in a haploid genome is 0.01×*TL*, where *L* is the total genome length (taken to be 3435cM [38]). Under a hybrid-isolation model and assuming a genome-wide ancestry proportion of z, a fraction of *2×z(1−z)* of the recombination events occurs between two haplotypes of opposite ancestry and thus leads to transitions in ancestry. When we count the number of ancestry blocks in the real data, we do not observe recombination events that occur between two haplotypes of the same ancestry. Hence the expected number of ancestry switches in a diploid genome is *B = (2×2×0.01)×TL×z(1−z)*, and each ancestry switch creates one additional ancestry block. When there is no ancestry switch in a genome, the number of ancestry blocks is defined to be the same as the number of chromosomes. Therefore, for each specific time of admixing, *T*, we computed the expected number of ancestry blocks as *B+2×22*, with the genome-wide ancestry proportion, *z*, varying from 0 to 1 at 100 equally spaced grid points. Each curve in Figure 2D shows the expected number of ancestry blocks as a function of admixture proportions for a specific admixing time. The estimated numbers of European and non-European ancestry blocks from the Mexican individuals were tallied and compared to the expected values. To assess the impact of uncertainty associated with estimating the number of ancestry blocks, we note that errors in estimating locus-specific ancestry often create very short ancestry blocks. Hence, we simulated admixed genomes according to the hybrid-isolation model, but removed extremely short blocks (segments with <10 SNPs) from both simulated genomes and real data. The estimated admixing time remained the same under this alternative analysis, suggesting the estimated admixing time is relatively robust. The hybrid-isolation model was chosen because of the mathematical simplicity; under a more realistic continuous gene-flow model, the estimated times of admixing should be interpreted as an approximation of average admixing time, weighted by the relative level of gene-flow in each generation.

### Substructure within the European and Indigenous American component of the genome

To assess the sub-continental population structure, each Mexican genome was partitioned into three non-admixed genomes, by masking (i.e. setting to missing) alleles from all but one ancestral population. In other words, the European component of a Mexican's genome (Mex^EUR^) was derived by treating as missing all alleles whose origins were inferred as African or Indigenous American. For within European analysis, we applied ssPCA on the dataset consisting of Mex^EUR^ (including both Mexico City and HapMap samples), 88 HapMap CEU and 176 European individuals from four cities: Dublin (Ireland), Warsaw (Poland), Rome (Italy) and Porto (Portugal). Because of the limited number of informative haplotype segments, Mexican individuals with less than 25% European ancestry were excluded from this analysis. In an analogous fashion, we analyzed the Mexican component of the genome, Mex^AMR^, along with 129 indigenous Indigenous American individuals representing 8 populations (Table S1). Because the African ancestry is low in both Mexican cohorts (3% and 5%, respectively), we did not analyze the within-Africa population structure.

### Detecting signature of selection

or all SNPs with frequencies between .05 and .95 in the respective populations, iHS was calculated following Voight et al. [8] with two modifications. First, haplotype homozygosity scores for a core SNP is computed on the subset of haplotypes in which the core SNPs are derived from a specific population. If a haplotype is truncated because of an ancestry change, the haplotype beyond the ancestry switch point is considered different from all other haplotypes in the corresponding interval. We have also considered an alternative strategy in which only haplotypes that do not have an ancestry change within 400 SNPs from the core SNPs are included in the calculation; the results are virtually identical. Second, instead of binning SNPs by the inferred ancestral allele frequencies and calculating the standard deviation of iHS in each bin, we used a quantile regression to estimate the 25th- and 75th-percentile of the empirical null distribution as a function of the minor allele frequency. The raw iHS scores were then normalized by the estimated inter-quartile range within each chromosome; the resulting standardized iHS scores fit a standard normal distribution well. To define regions that may harbor recently adaptive alleles, we seeded a region by a window of 50kb around a SNP with extreme iHS scores; we then successively scanned to the left and to the right, 50kb a time, merging neighboring regions in which at least one SNPs has an |iHS|>2.5 (which represents the 99th-percentile of the scores); finally, the top 10 list in Table 1 requires that at least 10% of the SNPs in the region have |iHS|>2.5. The proportion of SNPs with high |iHS| was the criterion used by Pickrell et al. [9]. It has been suggested that genome-wide and locus-specific ancestries may show particular poor correlation at loci under selection compared to neutrally evolving loci [39]. We did not observe such a trend at loci with the highest iHS scores in either MEX^AMR^ or MEX^EUR^.

## Supporting Information

Figure S1 Comparison of genome-wide ancestry proportions estimated by *frappe* and by averaging SABER+ locus-specific ancestry across all markers: yellow = Indigenous American, red = European, blue = African.
(TIFF)

Figure S2 Simulation experiments for assessing the robustness of ssPCA in the presence of uncertainty associated with the inference of locus-specific ancestry. The mean confusion fraction for separating Northern and Southern European substructure (y-axis, averaged over 10 independently simulated datasets) increases with the error rate in the putative virtual genome (x-axis). Arrow indicates the mean confusion fraction when ssPCA is applied to virtual genomes inferred using SABER+ (ξ = 1.58%).
(TIFF)

Figure S3 PC1 versus PC3 separate Mex^AMR^ from Pima individuals.
(TIFF)

Table S1 Individuals and markers used for analyses.
(PDF)
