# Supplementary figures and images for: Ancestral Components of Admixed Genomes in a Mexican Cohort
Source: PLoS Genet. 2011 Dec 15;7(12):e1002410. doi: 10.1371/journal.pgen.1002410 (PMC3240599; doi:10.1371/journal.pgen.1002410)

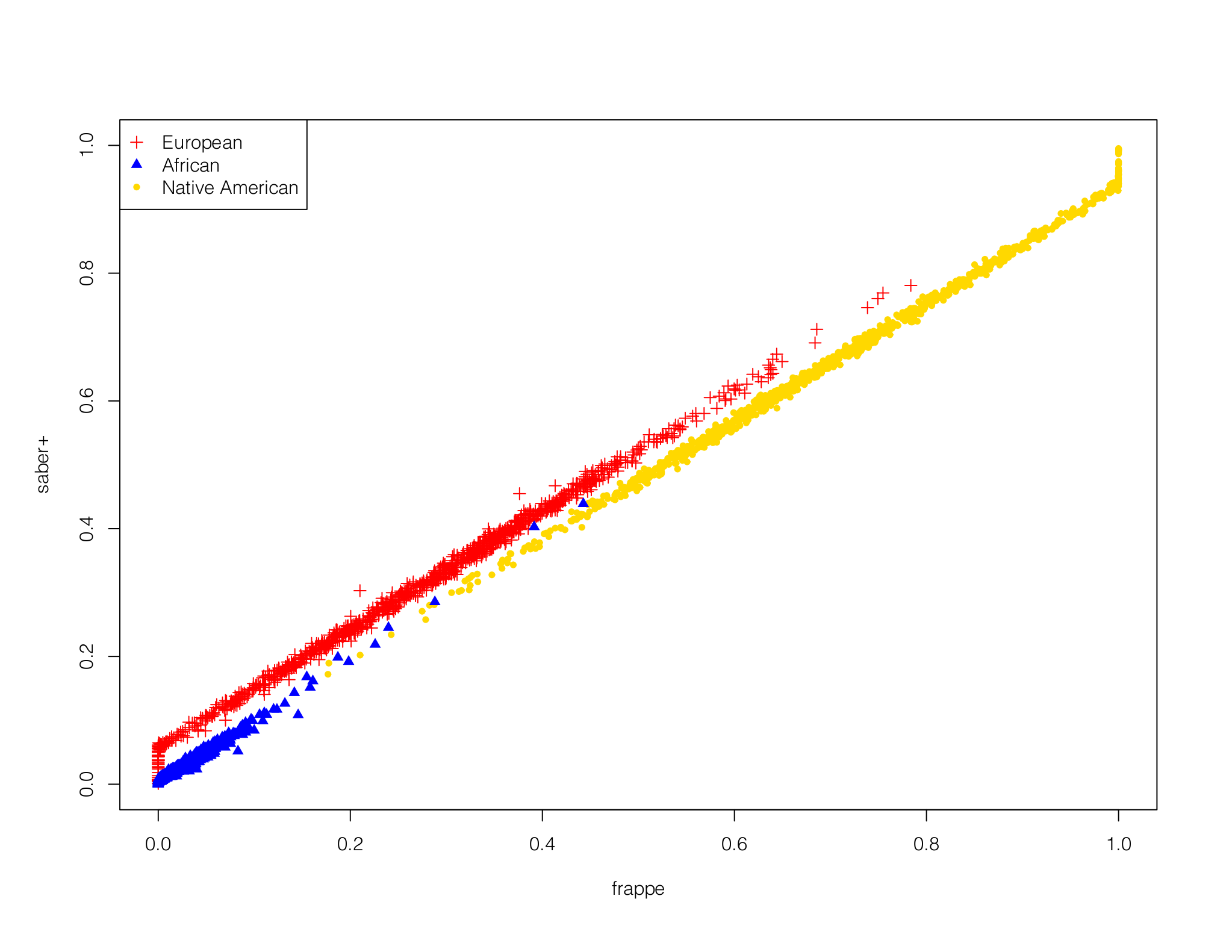

Supplement: Figure S1 — Comparison of genome-wide ancestry proportions estimated by frappe and by averaging SABER+ locus-specific ancestry across all markers: yellow = Indigenous American, red = European, blue = African. (TIFF) [file pgen.1002410.s001.tiff]

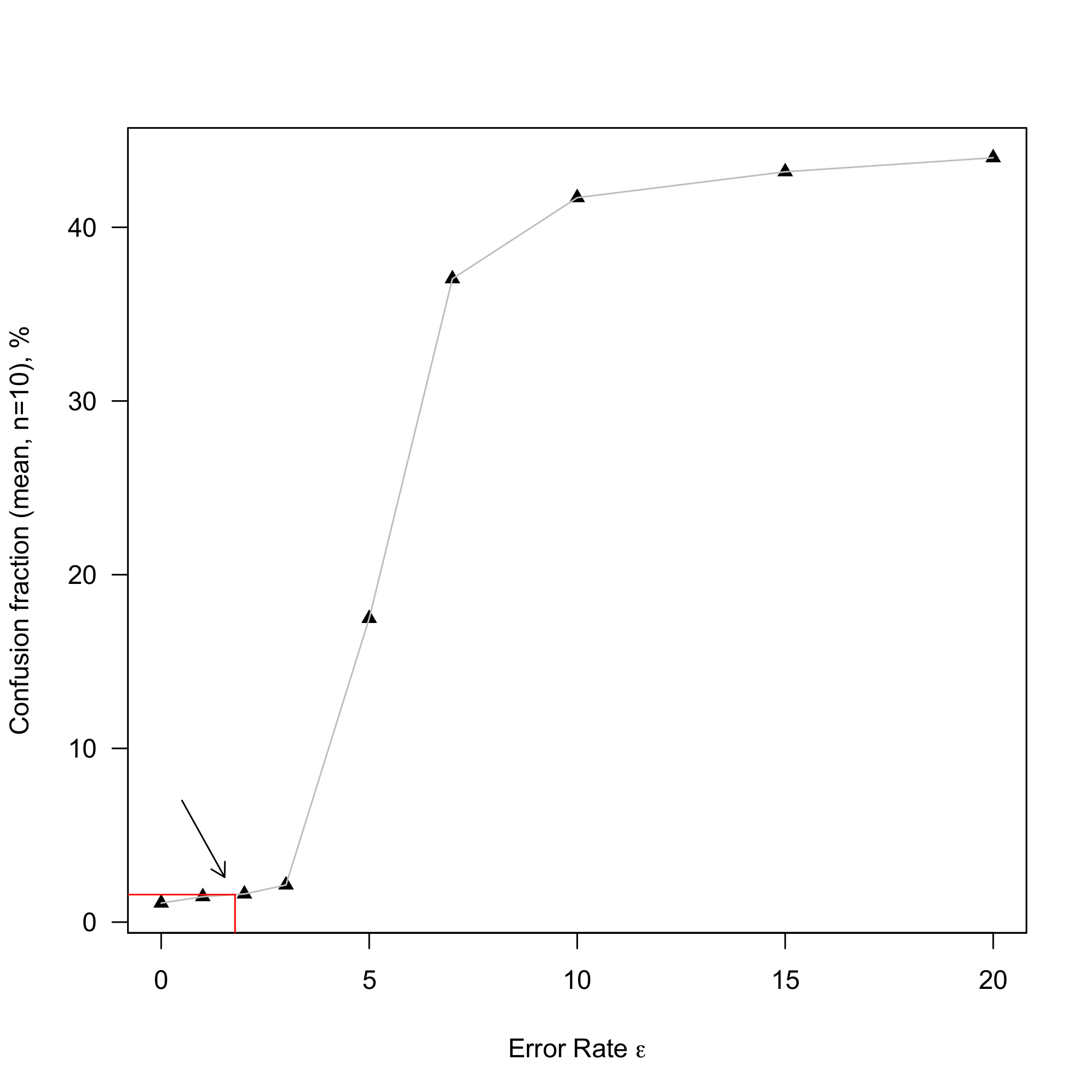

Supplement: Figure S2 — Simulation experiments for assessing the robustness of ssPCA in the presence of uncertainty associated with the inference of locus-specific ancestry. The mean confusion fraction for separating Northern and Southern European substructure (y-axis, averaged over 10 independently simulated datasets) increases with the error rate in the putative virtual genome (x-axis). Arrow indicates the mean confusion fraction when ssPCA is applied to virtual genomes inferred using SABER+ (ξ = 1.58%). (TIFF) [file pgen.1002410.s002.tiff]

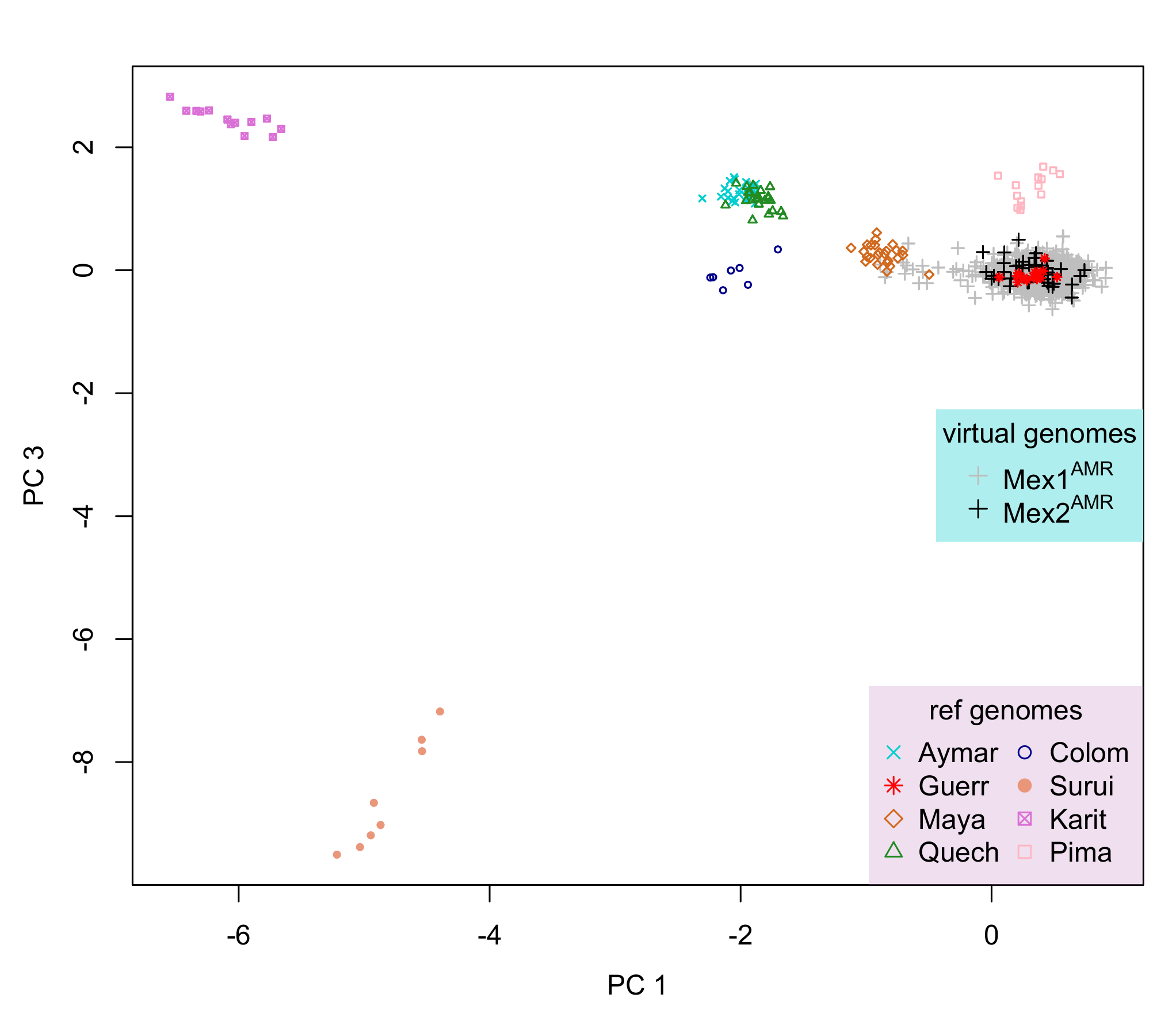

Supplement: Figure S3 — PC1 versus PC3 separate MexAMR from Pima individuals. (TIFF) [file pgen.1002410.s003.tiff]
