# Supplementary material for: Ancestral Components of Admixed Genomes in a Mexican Cohort
Source: PLoS Genet. 2011 Dec 15;7(12):e1002410. doi: 10.1371/journal.pgen.1002410 (PMC3240599; doi:10.1371/journal.pgen.1002410)
Supplement: Table S1 — Individuals and markers used for analyses. (PDF) [file pgen.1002410.s004.pdf]

Table S1: Individuals and markers used for analyses.

Analysis	Individuals (sample size)	# of SNPs
Frappe, PCA for genome-wide ancestry;<br>Estimation of locus-specific ancestry	CEU (88); YRI (100);<br>MEX1 (984); MEX2 (46)	482906
Within European PCA	Irish (43); Polish (45); Portuguese (43); Italian (45); $MEX1^{EUR}$ (633); $MEX2^{EUR}$ (46)	162382
Within Indigenous American PCA	Indigenous Americans (129); $MEX1^{AMR}$ (933); $MEX2^{AMR}$ (26)	162382
Signature of selection	MEX1 (957)	482906
